# Socio-economic outcome after blunt orthopaedic trauma: Implications on injury prevention

**DOI:** 10.1186/1754-9493-5-9

**Published:** 2011-05-13

**Authors:** Roman Pfeifer, Philipp Lichte, Boris A Zelle, Nicola-Alexander Sittaro, Anna Zilkens, Jason R Kaneshige, Hans-Christoph Pape

**Affiliations:** 1 University of Aachen Medical Center, Department of Orthopaedic Trauma, Pauwelsstraße 30, 52074 Aachen, Germany; 2 Department of Orthopaedics, University of Pittsburgh Medical Center, 3471 Fifth Avenue Suite 1010, Pittsburgh, PA 15213, USA; 3 Hannover Life RE-Insurance, Medical Division, Karl-Wiechert-Allee 50, 30625 Hannover, Germany; 4 Parkland Health and Hospital Systems Department of Orthopaedic Surgery, Dallas, USA

## Abstract

**Background:**

Several large studies have identified factors associated with long-term outcome after orthopaedic injuries. However, long-term social and economic implications have not been published so far. The aim of this investigation is to study the long-term socio-economic consequences of patients sustaining severe trauma.

**Methods:**

Patients treated at a level one trauma center were invited for a follow-up (at least 10 years) examination. There were 637 patients who responded and were examined. Inclusion criteria included injury severity score (ISS) ≥ 16 points, presence of lower and upper extremity fractures, and age between 3 and 60 years. Exclusion criteria included the presence of amputations and paraplegia. The socio-economic outcome was evaluated in three age groups: group I (< 18 years), group II (19 - 50 years), and group III (> 50 years). The following parameters were analyzed using a standardized questionnaire: financial losses, net income losses, pension precaution losses, need for a bank loan, and the decrease in number of friends.

**Results:**

510 patients matched all study criteria, and breakdown of groups were as follows: 140 patients in group I, 341 patients in group II, and 29 patients in group III. Financial losses were reported in all age groups (20%-44%). Younger patients (group I) were associated with less income losses when compared with other groups (p < 0.05). Financial deterioration was more frequently reported in age group II (p < 0.05). Social consequences (number of friends decreased) were predominantly stated in patients younger than 18 years old (p < 0.05).

**Conclusions:**

Economic consequences are reported by polytraumatized patients even ten or more years after injury. Financial losses appear to be common in patients between 19 and 50 years. In contrast, social deprivation appears to be most pronounced in the younger age groups. Early socio-economic support and measures of injury prevention should focus on these specific age groups.

## Background

Polytrauma can permanently impact a person's quality of life and satisfaction. A limited functional state may cause vocational disabilities, and lead to financial dependence on social programs. Financial losses and a restricted role in society have a lasting impact on the individual, family, and on society. Long-term follow up investigations have shown that traumatized individuals with brain and spinal cord injuries more frequently demonstrate severe disabilities after polytrauma [1,2]. Large long-term investigations report that lower extremity injuries below the knee joint, and articular fractures have a significant impact on functional long-term recovery [1,3-9]. Numerous investigations identified further non-injury-related factors influencing long-term outcome, quality of life, and life satisfaction [10-12]. Gender, education and income are significant sociodemographic parameters associated with long-term outcome [12]. Others underline the role of post-injury professional life, depression, physical functioning, and pain as factors associated with degree of satisfaction [5,10,13].

There are no studies published so far analyzing the long-term social and economic consequences in patients who sustain polytrauma. Therefore, the aim of this investigation is to analyze the socioe-conomic outcome in polytraumatized patients more that 10 years after the traumatic event.

## Methods

The investigation was designed as a cohort study. The study protocol was approved by the local Institutional Review Board and written informed consent was obtained by all participants.

Patients with multiple injuries treated at a level one trauma center were re-evaluated to obtain functional and socio-economic long-term outcome data following polytrauma. A retrospective chart review of all patients treated between January 1, 1973 and December 31, 1990 was performed. Patients who met the inclusion and exclusion criteria were invited for a physical examination and completed standardized questionnaires. Details of the recruitment process and avoidance of biases in this study were previously published [9]. Using this database (total *n *= 637 Patients), patients with upper and lower extremity fractures were identified.

Patients were included in the study if they met the following criteria:

• Multiple blunt orthopedic injuries

• Injury Severity Score (ISS) ≥ 16

• Fractures of the upper and lower extremity

• Age at the time of injury between 3 years and 60 years

• Minimum follow-up of at least ten years

The following exclusion criteria were used:

• Patients with amputations of upper or lower extremity

• Patients with paraplegia

Group Distribution:

To assess the age related differences in regard to socio-economic outcome, patients were divided into three age groups:

• Age Group I: < 18 years at time of injury

• Age Group II: 19-50 years at time of injury

• Age Group III: > 50 years at time of injury

Demographic and clinical data extracted from the patient chart included the patients age, gender, cause of injury, follow-up period, and injury severity measured by ISS [14]. The socioeconomic outcomes at follow-up were evaluated using a standardized self-administered patient questionnaire, which included the following questions:

• "Do you still have financial losses as a consequence of prior injury?"

• "Do you still have losses of your net income as a consequence of prior injury?"

• "Do you have losses of pension precaution as a consequence of prior injury?"

• "Did you have to take out a bank loan due to financial consequences of prior injury?"

• "Did the number of your friends decrease as a consequence of prior injury?"

Statistic analysis was performed using SAS^® ^statistical software, V9.1.3 procedure LOGISTIC (SAS Institute, Cary, NC, USA). Statistical significance was set at a p-value of < 0.05. Descriptive statistics were performed to describe the sociodemographic and injury-related characteristics of the sample. Differences between the groups of patients on socio-economic outcomes at 10+ years follow-up were tested using Chi-Square analyses and Kruskal Wallis tests for non-normally distributed continuous variables, and Analysis of Variance (ANOVA) was used when normal distribution was present.

## Results

Of the of 637 recorded trauma patients, 552 patients were identified to have extremity injuries. A further 42 patients were excluded due to the presence of upper (*n *= 8) or lower (*n *= 17) extremity amputations or paraplegia (*n *= 17). The remaining 510 patients matched all study criteria and were included. Among those were 81 patients with upper extremity injuries, 215 patients with lower extremity injuries, and 214 patient's with combined trauma of the upper and lower limb. At the time of injury, 140 patients were less than 18 years old (age group I), 341 patients between 19 and 50 years old (age group II), and 29 patients were older than 50 years (ager group III). Table 1 provides further details regarding demographic characteristics. No difference in injury severity was found between the age groups.

**Table 1:** Demographic characteristics of the study population (*n = *510)

	Age Group I 0-18 years	Age Group II 19-50 years	Ager Group III > 50years	p value
Number of Patients	140	341	29	-
Mean Age at Time of Injury	16	23	51	-
Mean Age at Follow Up	34	44	68	-
Male	69.3%	77.1%	62.1%	ns
Mean ISS	18.2	20.7	20	ns
Follow Up in Years	19	16	15	ns

**Figure 1:**
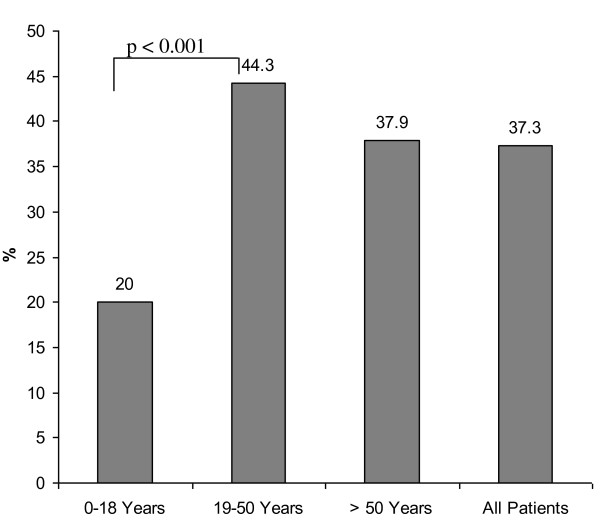
Frequency of financial losses in each age group

**Figure 2:**
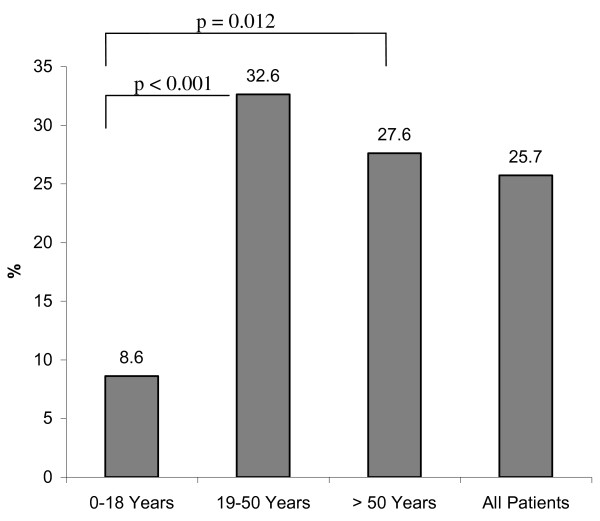
Frequency of net income losses in each age group

**Figure 3:**
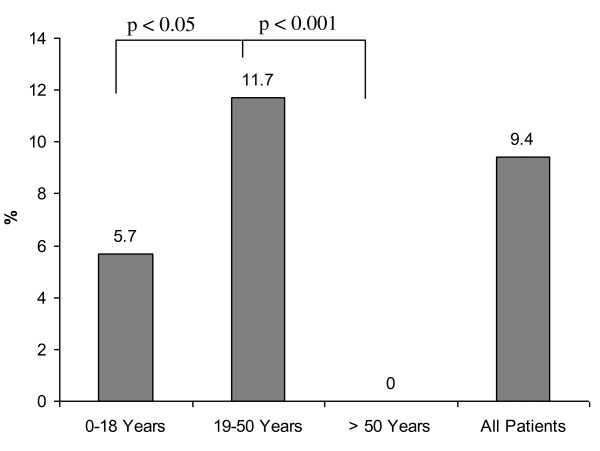
Frequency of pension precaution losses in each group

**Figure 4:**
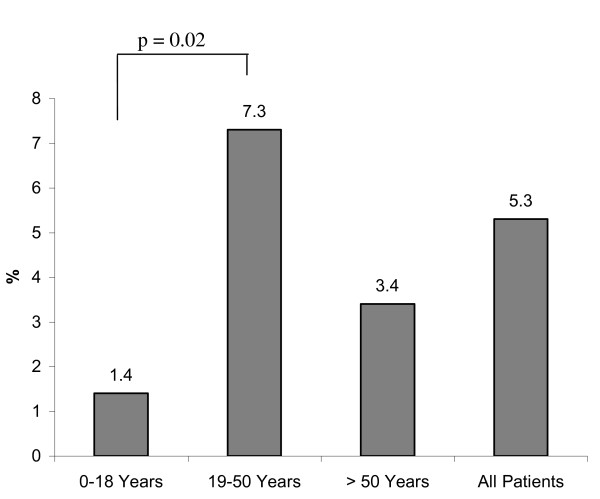
Frequency of patients borrowing a loan in each age group

Figure 1 shows the percentage of patients with financial losses in each age group. At the time of examination, financial losses were reported in 20% of patients in age group I (< 18 years), in 44.3% of patients in age group II (19-50 years), and 37.9% in age group III (> 50 years). Statistical significance was found between age group I and age group II (*p *< 0.0001). Differences between groups I and II (p = 0.06) and groups II and III (p = 0.4) did not reach statistical significance. At follow up, deterioration of net income was reported in 25.7% of the study population after their injuries. In group I, a loss of net income was less frequently reported compared with other groups (Figure 2; 8.6% vs. 32.6%, *p *= < 0.0001; 8.6% vs. 27.6%, *p *= 0.012; 32.6% vs. 27.6%; *p *= 0.22). In addition, 9.4% of the entire study population reported losses of pension precaution. Patients in age group II reported more frequent losses of pension precaution (Figure 3, 5.7% vs. 11.7%, *p *= 0.05; 5.7% vs. 0%, *p *= 0.9; 11.7% vs. 0%, *p *< 0.0001). In regard to financial debts, 1.4% of patients in age group I, 7.3% of patients in age group II, and 3.4% of patients in age group III required a bank loan after injury (Figure 4; 1.4% vs. 7.3%, *p *= 0.02; 1.4% vs. 3.4%, *p *= 0.4; 7.3% vs. 3.4%, *p *= 0.5).

**Figure 5:**
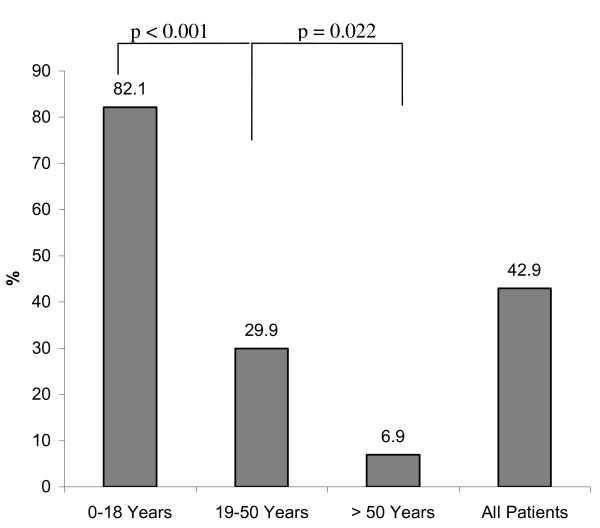
Frequency of patients reporting decreased number of friends in each age group

To assess the social consequences of severe injury, patients were asked whether their number of friends decreased following injury. We observed that younger patients (age group I) more frequently stated that their number of friends has decreased. At follow up, 82.1% of patients younger than 18 years at the time of injury reported that their number of friends has decreased. Other age groups (age group II: 29.9% and age group III: 6.9%) reported loss of friendship less frequently upon follow-up (Figure 5; 82.1% vs. 29.9%, *p *< 0.0001; 82.1% vs. 6.9%, *p *< 0.0001; 29.9% vs. 6.9%, *p *= 0.022).

## Discussion

Numerous long-term investigations have been conducted in order to evaluate the functional recovery of polytrauma patients [3-9]. Persistent functional disorders, disabilities, and chronic pain are common findings. The pivotal role of psychosocial factors and goals such as recovery to pre-injury functional status, social reintegration, and return to work have gained more attention in trauma care [3-9].

Our main findings can be summarized as follows: 1. Financial and net income losses were reported in all age groups. 2. Patients between 19 to 50 years (age group II) reported more frequent financial and net income losses and were more often in debt. 3. Negative social consequences (number of fiends reduction) were more frequently stated in the youngest age group.

The following limitations have to be considered when interpreting the results of our study. (1) The retrospective evaluation of the data represents a drawback of this study. (2) Subjective parameters (financial losses and loss of friends) were chosen as outcome measures. Financial and net income losses were not quantified. Thus, any financial losses reported by patients were registered in our study. (4) The absence of a control group and a relatively small patient number in the age group III (*n = *29) are further drawbacks of this study. (5) We are unable to generalize our conclusions since individuals only with upper and lower extremity injuries were included in this investigation, while excluding patients with amputations and paraplegia. These injuries are known to be particularly associated with inferior outcomes as reported by the LEAP study group [5,10,15]. Nevertheless, we feel that the long follow-up and large group size (Group I and II) are strengths of our investigation.

In addition, it must be noted that the consequences of the traumatic brain injury on long-term socio-economic outcome were not studied in this investigation. Using the same database our study group could demonstrate that patients who sustained a traumatic brain injury were more like to be female, younger in age and had a greater number of upper extremity injuries [16]. These patients were associated with inferior long-term psychological functioning and higher rates of chronic pain [16].

In our study, patients 18 years of age or younger less frequently reported financial losses after injury. This may reflect an improved physical recovery from trauma. Younger patients are more capable to adapt to life changes and to adjust to their disabilities. Several groups have demonstrated evidence of age-related differences in recovery after severe injury [2,13]. At follow up, the majority of pediatric polytrauma patients demonstrated lower rates of disability and the quality of life did not differ compared to healthy reference populations [2].

In addition, early return to productivity in this age group (≤ 18 years) might play a crucial role as well. Studies have identified lower age as an independent predictor of return to work [17,18]. Long-term (1 to 7 years) follow up investigations have reported a return to work rate of 52% to 64% [6,19,20]. Even those who returned to work reported limitations in performing jobs, or chose a less physically demanding position [6]. Severe extremity injuries and chronic pain are known factors influencing employment status and socio-economic outcome [6,13]. However, numerous investigations point out a weak correlation between physical function and the rate of return to work [21-23]. MacKenzie et al. reported that socioe-conomic characteristics (higher education, higher income, higher social support) and job-related factors (job flexibility, employment in jobs with low physical demands) were associated with higher rates of return to work at 12 months after severe lower limb injury [13]. Moreover, authors also introduced the importance of self-efficacy in the rehabilitation process [6]. High self-efficacy was among the strongest predictors of return to work. The authors assumed that patients with low self-efficacy are more likely to be disengaged from the rehabilitation and recovery process [6].

In contrast to economic outcome, social consequences of polytrauma are more often reported in younger patients and are less frequent in patients older than 50 years old. Advantages of a supportive family and social network on post-injury outcome and life satisfaction were pointed out by several authors [21,24,25]. This finding may be explained by the fact that long hospital stays and prolonged rehabilitation processes may result in limited contact with friends. Furthermore, functional disabilities, cognitive problems and economic sequelae may interfere with leisure activities. It was demonstrated that a number of students failed to pass classes or had to change schools after trauma [2]. Consequently, they were taken out of their familiar environment and circle of friends. In contrast, we assume that elderly patients are less flexible due to their close link to family and have a stable circle of friends. Therefore, loss of friendship appears to be less relevant for elderly patients in our study.

## Conclusions

In conclusion, traumatic events yield long-term significant financial and net income losses in all age groups, especially in those aged between 19 and 50 years. Social deprivation appeared to be most pronounced in the youngest population. This study underlines that polytrauma is a life-altering event leading to prolonged morbidity, lasting disability, and immense socioe-conomic burdens on the affected individual, their families, and society. Measures of injury prevention should focus on these particular age groups to analyze their consequences.

## Competing interests

This study was supported by Hannover-Re Insurance. There are no further conflicts of interest.

## Authors' contributions

All authors were involved in the research project and approved the final version of the manuscript. HCP: He made a substantial contribution to conception and design, and gave a critical and final approval. BZ; NAS: Authors collected the data, were responsble for acquisition of funding and were involved in drafting and revising of the manuscript. RP; PL; AZ; JRK: Authors have collected the data and made the analysis and interpretation of these data. Moreover, authors also made a draft of the manuscript and revision. All authors read and approved the final manuscript.
